# Severe Hair Dye Anaphylaxis Caused by Toluene‐2,5‐ Diamine Identified by Positive in Vitro Tests

**DOI:** 10.1111/cod.70159

**Published:** 2026-04-22

**Authors:** Eglė Janušonytė, Thomas Harr, Pierre Piletta, Sophie Vandenberghe‐Dürr

**Affiliations:** ^1^ Department of Dermatology and Venereology Geneva University Hospitals Geneva Switzerland; ^2^ Department of Clinical Immunology and Allergy Geneva University Hospitals Geneva Switzerland

**Keywords:** anaphylaxis, basophil activation test, hair dye allergy, lymphocyte transformation test, p‐phenylenediamine, toluene‐2,5‐diamine

## 1 Case Report

We report a case of a severe allergic reaction following hair dye application in a 60‐year‐old woman with a history of liver transplantation in 2003, maintained on tacrolimus 3.5 mg. The patient was known for multiple allergies, including peanut, oral allergy syndrome, and anaphylaxis to vancomycin and influenza vaccination. Ten minutes after the application of a black‐coloured hair dye (Syoss Permanent, Henkel AG & Co. KGaA, Germany) in 2024, the patient developed diffuse urticarial lesions, followed by nausea and tingling sensations at the tip of the tongue, lingual edema, dyspnea, and dysphagia. No hemodynamic alterations or bronchospasm were present. Serum tryptase was 19.5 μg/L, confirming mast cell degranulation (baseline serum tryptase was 6.2 μg/L). Her condition rapidly improved upon treatment with intramuscular administration of adrenalin, methylprednisolone, and antihistamines.

**FIGURE 1:**
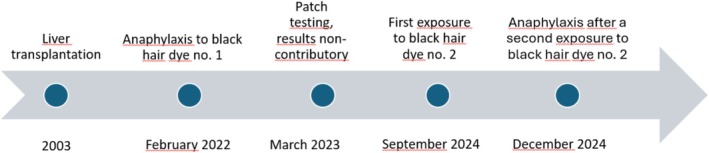
Timeline of anaphylaxis related events.

Review of the patient's history revealed that she had tolerated the same hair dye 4 months prior. Two years prior however, she had experienced a similar anaphylactic reaction to a different black hair dye containing toluene‐2,5‐diamine (Garnier Olia, L'Oréal, Belgium) with lingual edema, dysphonia, and dysphagia (Figure 1). Skin tests were performed at that time under strict medical monitoring. Standard patch tests with a baseline and hairdressing series (supplied by Chemotechnique Diagnostics, Vellinge, Sweden) using IQ Ultra patch test chambers (Chemotechnique Diagnostics), as well as semi‐open patch testing with the patient's personal dyes diluted with 10% aqua, were all negative, both at immediate (20 min) and delayed reading (96 h).

After the second reaction, the patient opted for further prick testing. We performed prick‐to‐prick skin tests using toluene‐2,5‐diamine 1% petrolatum and p‐phenylenediamine 1% petrolatum. Appropriate positive (histamine, codeine) and negative (NaCl) controls were included: the histamine control was positive (+7 mm) and the codeine and saline control negative. No positive reactions to the tested substances were observed at 20 min. In contrast, the basophil activation test (BAT, ADR‐AC, Switzerland) showed strong positivity for toluene‐2,5‐diamine, as well as borderline positive reactivity for p‐phenylenediamine (Table 1). The lymphocyte transformation test (LTT, ADR‐AC, Switzerland) revealed IL‐13 reaction positivity for p‐phenylenediamine, but negative results for toluene diamine (Table 2).

**TABLE 1:** Basophil activation test results (IL‐3—interleukin 3, CD63—cluster of differentiation 63, SI—stimulation index, CD203c—cluster of differentiation 203c).

Stimulant	Concentration μg/ml	With IL‐3	With IL‐3	Without IL‐3	Without IL‐3	Without IL‐3	Without IL‐3
		CD63	SI	CD63	SI	CD203c	SI
p‐Phenylenediamine	1	5.6%	**2.8**	5.3%	**2.4**	6.9%	**3.0**
p‐Phenylenediamine	0.2	2.4%	1.2	3.4%	1.5	3.8%	1.6
p‐Phenylenediamine	0.05	2.5%	1.2	1.4%	0.6	0.9%	0.4
Toluendiamine	1	14.1%	**7.0**	14.6%	**6.5**	22.4%	**9.6**
Toluendiamine	0.2	10.0%	**4.9**	14.2%	**6.4**	22.7%	**9.7**
Toluendiamine	0.05	4.1%	**2.0**	4.7%	**2.1**	11.3%	**4.8**
Control							
Buffer		2.0%		2.2%		2.3%	
Anti‐IgE		51.3%		51.6%		48.1%	
N‐formylmethionine‐leucyl‐phenylalanine (Fmlp)		68.3%		71.4%		56.3%	

*Note:* Basophil activation test results (IL‐3 ‐ interleukin 3, CD63 ‐ cluster of differentiation 63, SI ‐stimulation index, CD203c ‐ cluster of differentiation 203c) with strong positivity for toluene‐2,5‐diamine, as well as borderline positive reactivity for p‐phenylenediamine.

**TABLE 2:** Lymphocyte transformation test results (IL‐5—interleukin 5, IL‐13—interleukin 13, IFN‐γ—interferon gamma, GzB—granzyme B, GL—gliadin).

Substance	Il‐5	IL‐13	IFN‐*γ*	GzB	GL
p‐Phenylenediamine	Negative	Positive	Negative	Negative	Negative
0.05 μg/mL	1.00	3.43	0.52	0.50	0.44
0.2 μg/mL	**25.68**	**20.47**	0.52	1.83	0.55
1 μg/mL	1.20	6.80	0.52	0.41	0.68
Toluendiamine	Negative	Negative	Negative	Negative	Negative
0.05 μg/ml	1.00	1.00	0.52	0.34	0.54
0.2 μg/ml	1.00	1.00	1.00	0.30	0.51
1 μg/ml	1.00	1.00	0.52	0.45	0.50
Control	Positive	Positive	Positive	Positive	Positive
Pokeweed mitogen	159.05	1188.40	1566.58	810.54	7.18
Tetanus toxoid	14.65	22.38	19.77	35.12	0.61

*Note:* Lymphocyte transformation test results (IL‐5 ‐ interleukin 5, IL‐13 ‐ interleukin 13, IFN‐γ ‐ interferon gamma, GzB ‐ granzyme B, GL ‐ gliadin), revealing IL‐13 reaction positivity for p‐phenylenediamine,but negative results for toluene diamine.

## 2 Discussion

P‐phenylenediamine and toluene‐2,5‐diamine are common causes of allergic contact dermatitis, sometimes necessitating systemic treatment. Far less known is the fact that these hair dyes can also cause anaphylactic reactions, with the consequence that a missing diagnosis of a potential immediate type allergy can lead to re‐exposure with the culprit hair dye [1, 2]. Here, we were able to show that the diagnosis can be established with in vitro testing by BAT and LTT despite negative skin tests and systemic immunosuppressive treatment with tacrolimus. BAT and LTT should therefore be considered in patients with immediate type reactions to hair dye, even in immunosuppressed patients. In our case, the clinical presentation along with the significant tryptase elevation and the positive BAT for toluene‐2,5‐diamine strongly suggests an IgE (or IgG)‐mediated anaphylactic reaction [3].

LTT are usually used in the workup of delayed type drug‐induced hypersensitivity reactions. The positive LTT, with particular elevation of IL‐13, points toward an antigen‐specific reaction, rather than a non‐specific basophil activation, and indicates the presence of Th2‐type specific T cells. Of note, IL‐13 is a key Th2 cytokine exerting regulatory functions that amplify and sustain the allergic response [4]. IL‐13 directly promotes IgE class switching in B cells by activating the STAT6 signalling pathway [5]. Furthermore, IL‐13 overexpression primes systemic IgE hyperproduction by enhancing Th2 cell differentiation and amplifying B cell responsiveness to IL‐4/IL‐13 signals [6, 7]. This increased IgE production leads to increased binding to high‐affinity FcεRI receptors on mast cells and basophils, sensitizing cells to allergen‐induced degranulation and the subsequent release of anaphylactic mediators.

It should be noted that permanent hair dyes are complex formulations consisting of two parts: one containing colouring agents such as PPD and TDA, and another containing oxidizing agents such as hydrogen peroxide. Both parts may contain additional substances with allergenic potential. Accordingly, the clinical reaction could theoretically have been elicited by a component that was not specifically tested in our evaluation. Furthermore, oxidation of PPD and toluene‐2,5‐diamine during the dyeing process generates reactive intermediates and by‐products that may contribute to sensitization. Ideally, testing should also have included the individual hair dye components and the mixed formulation to capture potential reactions to these additional substances. While we acknowledge that testing the complete formulations and their oxidation products would have added diagnostic certainty, the strong and specific BAT positivity for TDA, the borderline‐only reactivity to PPD, and the patient's reactions to two different TDA‐containing products from different manufacturers together strongly suggest that TDA is the most likely causative agent.

The negative prick test despite positive BAT needs to be discussed. When the allergen is correctly identified, one would generally expect concordant skin and in vitro test results. However, we have to consider the patient's specific medication that might have an influence on the read out of the skin tests. Our patient was on tacrolimus, a calcineurin inhibitor known to suppress cutaneous mast cell reactivity and attenuate skin test response. In our case, the histamine mediated skin prick test was positive, indicating functional H1‐receptors in the skin tissue. In contrast, the codeine control depends on resident mast cells releasing endogenous histamine after stimulation; a negative response therefore points to decreased mast‐cell density and/or impaired degranulation capacity, which may contribute to the difference between skin and in vitro testing. Tacrolimus is a known suppressor of mast cell activation and histamine release acting probably via the MRGPRX2 receptor [8, 9]. Therefore, we were not astonished to see the difference between a positive histamine reaction on the skin induced by exogenous histamine and a lack of response to codeine as well as the suspected culprits due to a lack of mast cell activation by tacrolimus.

Additionally, petrolatum‐based preparations, while standard for patch testing, may not be optimal vehicles for prick testing with these compounds. BAT, by directly assessing basophil reactivity ex vivo, bypasses these cutaneous confounders and may therefore offer greater sensitivity in this clinical context.

To the best of our knowledge, this is the first documented case of IgE (or IgG)‐mediated hair dye anaphylaxis with positive results in both BAT and LTT. Potential cross‐reacting hair dyes should be included in the testing to increase patient safety.

Written informed consent was obtained from the patient for the publication of this manuscript, including any accompanying clinical information and images.

## Author Contributions

**Pierre Piletta:** writing – review and editing. **Eglė Janušonytė:** conceptualization, investigation, writing – original draft. **Sophie Vandenberghe‐Dürr:** writing – review and editing, conceptualization, investigation. **Thomas Harr:** writing – review and editing.

## Funding

The authors have nothing to report.

## Conflicts of Interest

The authors declare no conflicts of interest.

## Data Availability

Data sharing not applicable to this article as no datasets were generated or analysed during the current study.
